# Borax and Bromides in Epilepsy

**Published:** 1893-11-04

**Authors:** 


### New Drugs and Preparations.

[We should be glad to receive, at our Office, 428, Strand, London, W.C., from the manufacturers, specimens of all new preparations and drugs which may be brought out from time to time.]

### BORAX AND BROMIDES IN EPILEPSY.

The treatment of epilepsy has undergone great changes through advances in diagnosis. Exact methods of cerebral localisation, and the separation of various diseases which produce epileptiform attacks have reduced the number of true epilepsies. For these the chief and almost the sole remedy has been the bromides. In some cases they actually cure, in a majority they lessen the number and the force of the attacks. Certain patients take them for many years together with comfort and benefit, but there are disadvantages in many cases which cannot be overlooked. Even if we avoid producing an injurious influence on the heart by care in choosing our bromide compounds, we find in certain patients troublesome skin affections and great mental confusion almost amounting to a state of idiocy. Of course we may ask, would not these patients, had they been left to the destructive influence of their epileptic seizures, have fallen into an equally bad state of mental disintegration, varied by the dangers of constant seizures? Nor is it an answer to point out that by stopping the drug for a time they greatly improve mentally, for the possibility of such an improvement may be owing to their husbanded resources due to the long rest they have had. A patient, indeed, may be temporarily better after the explosive centres are exhausted by a seizure, though in the long run the attacks may steadily carry him down hill. Still, we may aim at combining freedom from seizures with a tonic effect on the healthy centres if possible. It is claimed that this can be often done by substituting borax for some of the bromide given, though it is not perhaps easy to tell what cases will or will not improve under it. Borax is by no means a new remedy, but has been little used since the discovery of bromides. In those instances where we have employed it the results have been so far satisfactory, but long experience and a clue to the type of disease where it is most active are necessary if borax is to come into common use.

Now, numbers of patients do go on with great comfort under very large doses of bromides, and many of the failures can be avoided by the use of the strontium salts. More than a ton of bromides are said to be employed per annum at the National Hospital for Epilepsy alone, and Dr. Feré believes that some cases which do not yield to ordinary doses improve greatly under huge doses, provided they are watched closely, weighed and examined frequently. In the Bicêtre Asylum there are twenty patients who take four or six drachms daily of bromide of potash and strontium. Some of them have gone on with it for years. In six the weight has increased, and in eleven permanent improvement has taken place. When such doses are being given, if loss of weight, mental depression, a low temperature, gastric, skin, or cardiac trouble occurs, the drug is at once stopped, and purgatives and diaphoretics are administered. Agostini, indeed, formulates a rule from his experiments that potassium bromide is harmless if not given in excess of 3·1 to 3·8 grains per kilogramme of the body weight, and if it be intermitted every third day. He insists that in these doses it stops or greatly diminishes the attacks, but warns us that we must, however, insure that the salt is pure and the patient's kidneys are healthy. Still, we doubt with Sequin whether occasional instances of idiosyncrasy will not be found where the drug is badly borne or useless. Most epileptics, too, can only be seen medically at intervals, and for them extreme doses are impossible. Feeble heart action, again, and the presence of any organic brain lesion diminishes the power to tolerate large doses, while young children bear very great ones. On the other hand, to insure that the physiological effect is obtained, the dose should be large enough to destroy the palate reflex, it must be given with great regularity, plentifully diluted, and if the attacks chiefly occur at one period in the twenty-four hours, the major part of the dose should be administered a few hours before that time. Arsenic and the alkaline waters aid in preventing acne, or we may, with Feré, use naphthol, and for ulcers in the mouth bismuth and naphthol.

The results of the strontium salts, so far as they have gone, appear very satisfactory, the symptoms of bromism are reduced to a minimum, and the therapeutic action is probably equal to that of the commoner salts. Thus Deny found that seven patients in six months had altogether 331 fits when taking potassium, and only 246 when placed upon strontium.

We can then sometimes extend the utility of bromides for difficult cases in these various ways by using strontium salts, by employing very large doses with due care, or by the use of adjuvants, but where bromides are unsatisfactory in spite of our best efforts, it is well to make trial of borax. Messrs. Russell and Taylor, at the National Hospital for Epilepsy, published some striking results in twenty cases where ordinary doses of bromides had little effect. They gave from 7 to 60 grains three times a day of the biborate of soda, and reduced the number of the attacks in a marked degree. Stewart believes that it has a special value for cases of nocturnal attacks, but Græme Hammond and William Alexander, of Liverpool, express disappointment in it when given alone. The latter, however, finds it invaluable when added to small doses of bromides, and has employed it extensively for years. The patients become bright, active, and energetic, very different to the miserable, sluggish, and introspective creatures they were when under large doses of bromide alone. At Maghull he never gives more than fifteen grains daily of bromide, except as a temporary sedative for violent patients. Twenty grains of biborate of soda and five grains of bromide of soda in syrup and water three times daily is his usual formula. Not only do the attacks diminish notably, but the mental troubles, the irritability, and the almost imbecile state of many of the patients improve in an extraordinary degree. The question arises whether the antiseptic effects of borax can in any way produce these results, just as Feré prevents bromide ulceration by giving naphthol. We need not suppose epilepsy to be due to microbes, but it is possible that the lowered vitality of its victims allows of putrefactive and similar changes ending in the production of toxines. In support of this view, it may be noted that Herter and Smith found in cases of *petit mal* a high percentage of uric acid excreted, and an excessive amount of the ethereal sulphates in the urine at the time of the attacks. While under antiseptic medicines the patients showed some improvement.

(*To be continued.*)

